# Does endolymphatic hydrops affect cochlear and vestibular nerve volumes? An MRI-based morphometric study

**DOI:** 10.3389/fneur.2026.1819407

**Published:** 2026-04-08

**Authors:** Anne R. J. Péporté, Joana Kostova, Gustav Andreisek, Fabian Schön, Franca Wagner

**Affiliations:** 1 Department of Radiology, Cantonal Hospital Frauenfeld, Frauenfeld, Switzerland; 2 Department of Radiology, Cantonal Hospital Münsterlingen, Münsterlingen, Switzerland; 3 Institute of Diagnostic and Interventional Radiology, University of Zurich, Zurich, Switzerland; 4 Department of Diagnostic and Interventional Neuroradiology, Cantonal Hospital Aarau, Aarau, Switzerland; 5 Department of Otorhinolaryngology and Head and Neck Surgery, University Hospital Bern, Bern, Switzerland

**Keywords:** cochlear nerve volume, endolymphatic hydrops, magnetic resonance imaging, Ménière disease, nerve morphometry, vestibular nerve volume

## Abstract

**Objective:**

To investigate the relationship between the severity of endolymphatic hydrops (EH) and volumetric changes in cochlear and vestibular nerves using high-resolution MRI, and to assess associations with clinical audiovestibular symptoms.

**Methods:**

A cohort of 108 patients with clinically and radiologically confirmed EH underwent 3 T MRI to obtain volumetric measurements of the cochlear and vestibular nerves. Nerve volumes were compared across EH grades using non-parametric statistics. Cochlear nerve volumes in affected and unaffected ears were compared in a subgroup with unilateral hearing loss. Associations between EH severity and symptoms (hearing loss, vertigo, tinnitus, aural fullness) were evaluated.

**Results:**

Cochlear nerve volumes did not differ significantly between EH grades (*p* = 0.057), with a nonsignificant tendency toward larger volumes in mild EH that did not survive correction for multiple testing. Vestibular nerve volumes did not differ significantly by vestibular EH grade (*p* = 0.64). Comparisons of cochlear nerve volume between affected and unaffected ears in patients with unilateral hearing loss also showed no significant differences (*p* = 0.56). EH severity was not significantly associated with prevalence of hearing loss, vertigo, tinnitus, or aural fullness.

**Conclusion:**

EH severity does not significantly affect cochlear or vestibular nerve volumes measurable by MRI, nor does it strongly correlate with prevalence of clinical symptoms. These findings indicate that volumetric MRI, while reproducible and feasible, may have limited sensitivity as an imaging biomarker of EH. This underscores the need to explore alternative MRI-based structural or functional markers in future multimodal and longitudinal studies.

## 1 Introduction

Endolymphatic hydrops (EH) involves excessive endolymphatic fluid accumulation within the membranous labyrinth of the inner ear and represents a pathological hallmark of primary [e.g., Ménière’s disease (MD)] and secondary hydropic ear disease. Patients with EH experience episodic vertigo, fluctuating hearing loss, tinnitus, and aural fullness (1, 2). However, the underlying mechanisms encompassing fluid dynamics (3), protein alterations (4), and potential neural degeneration (5) remain debated. Recent experimental models highlight hyperplastic epithelial growth over mere hydrostatic distension as a driver of EH, challenging classic pressure-based theories (6).

Traditionally, the diagnosis of EH was clinical, relying on symptomatology and audiometric findings, as direct visualization of the hydrops was not possible with earlier imaging techniques. High-resolution 3 T MRI with delayed post-gadolinium sequences now enables *in vivo* visualization and grading of EH, including vestibular-cochlear ratios, herniation and perilymphatic enhancement patterns relevant to MD (7–10). The detection of EH with MRI has been incorporated into diagnostic criteria for MD, including the latest guidelines from the Japan Society for Equilibrium Research (11) and recent classifications (12, 13).

Yet concerns over gadolinium risks—nephrogenic systemic fibrosis in renal impairment (14) and brain deposition with repeated use (15)—fuel demand for non-contrast MR imaging biomarkers. Even contrast-enhanced quantitative fluid volumetry demonstrates only modest correlations with hearing instability and faces inherent limitations in cochlear resolution and longitudinal reproducibility (16). While definite MD associates with more severe EH grades (17), cochlear volumetry remains constrained by spatial resolution (18). Alternative non-contrast approaches including perilymphatic signal changes (10) and vestibular aqueduct metrics (19, 20) have been proposed. The relationship between different vestibular tests in MD remains complex. Caloric testing and video-head impulse testing provide complementary rather than correlated information, with discrepancies observed in a substantial proportion of patients (21). Whether these functional test dissociations have an anatomical correlate at the vestibulocochlear nerve level remains uninvestigated using volumetric MRI.

This study represents the first large-scale, blinded 3D semi-automated volumetry of the cochlear and vestibular nerves across EH grades. No prior research directly links EH severity to vestibulocochlear nerve volumes, critical for validating morphometry as a biomarker amid multimodal EH imaging advances.

## 2 Materials and methods

### 2.1 Patient population

The study was approved by the Ethics Committee of Eastern Switzerland (Req-2024-01599 EKOS 24/236). The requirement for informed consent was waived. A retrospective review of our institutional digital database was carried out for delayed contrast-enhanced MRI scans of the inner ear for hydrops imaging between 01/2020 and 06/2025.

Patients with local and systemic diseases directly involving the temporal bone were excluded. Further exclusion criteria were severe motion artifacts and incomplete imaging. Inclusion criteria were age > 18 years and available high-resolution 3 T MRI scans encompassing the internal auditory canal (IAC) and including delayed post-gadolinium MRI sequences. MRI scans were excluded for the following reasons: poor imaging quality due to severe image distortion (*n* = 7), severe motion artifacts (*n* = 11), incomplete imaging (*n* = 2), and local/systemic diseases directly involving the temporal bone (*n* = 4). From an initial retrospective review of 132 potential participants yielding 144 MRI scans, a cohort of 108 patients with MRI-confirmed EH and documented audiovestibular symptoms previously described by Péporté et al. (22) met inclusion criteria: age >18 years and available high-resolution 3 T MRI covering the internal auditory canals and including delayed post-contrast sequences.

### 2.2 MRI acquisition

All participants underwent 3 Tesla MRI scanning (Siemens Magnetom Vida 3 Tesla, Philips Achieva 3 Tesla) using standardized protocols optimized to visualize inner ear structures, the vestibulocochlear nerve complex, and the endo- and perilymphatic spaces. Acquisitions included 3D high-resolution T2-weighted sequences and delayed post-gadolinium sequences (3D FLAIR and 3D T2w inversion recovery sequence 4 h post-contrast) (Table 1).

**Table 1:** MRI scanning parameters.

Sequence	Plane	Field of view (mm)	Echo time (ms)	Repetition time (ms)	Flip angle (°)	Slice thickness (mm)
T2	3D tra	150 × 150	shortest	1,500	90	0.8
FLAIR (4 h post-contrast)	3D tra	250 × 250	340	4,800	40	2
T2 inversion recovery (4 h post-contrast)	3D tra	75 × 150	177	6,000	180	1.4

Example of detailed scanning parameters of the specific sequences of our inner ear endolymphatic hydrops MRI protocol. 3 Tesla MRI Scanner (Philips Magnetom Achieva).

Tra, transverse; cor, coronal; mm, millimeters; ms, milliseconds.

### 2.3 MRI assessment and volumetry

#### 2.3.1 Volumetric measurement

**Figure 1:**
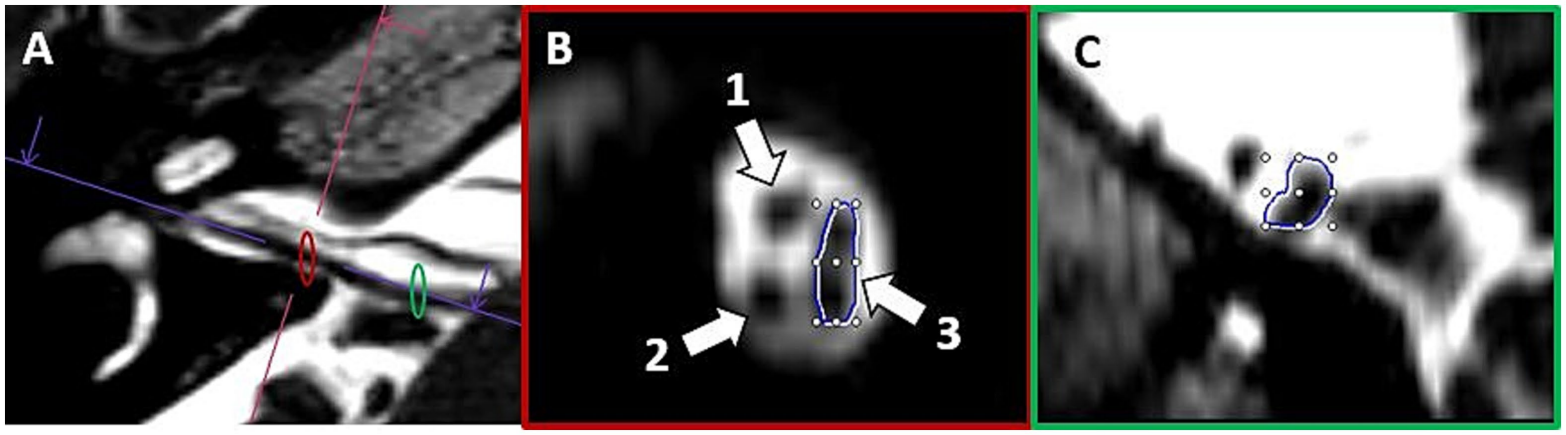
Example of volumetric measurement of the common vestibular nerve. Axial T2w SPACE image **(A)** at the level of the internal auditory canal showing the orientation of the common vestibular nerve and location of the oblique sagittal 0.8 mm multiplanar reformat from which neural volumetric measurements were obtained. The corresponding oblique sagittal reformatted image **(B)** at the fundus of the internal auditory canal is positioned lateral lateral to the cochlear aperture with the cochlear nerve (1), cochlear nerve (2), and the common vestibular nerve (3). Panels **(B,C)** show sections of the volumetric drawing of the common vestibular nerve, one example in the intrameatal segment (red ellipse) and one example in the cisternal segment (green ellipse).

Cochlear and vestibular nerve volumes were measured by AP (a board-certified neuroradiologist holding the European Diploma in Head and Neck Radiology, with more than 6 years’ experience in head and neck imaging), who was blinded to clinical data. Semi-automated segmentation software (Syngo.via software, Siemens Healthineers, version VB80D) was used for volumetric measurements. Measurements of the cochlear nerve and vestibular nerve complex volume were obtained bilaterally by axis-corrected measurement from the cerebellopontine angle to the internal auditory canal fundus. VOI regions were inserted throughout the course of the cranial nerves. The cumulative area of each MRI slice was then calculated by the software to obtain the nerve volume. The partial blurring seen at the margins of the reconstructed nerves (penumbra effect) was managed by delineating contours at the midpoint between the central low-signal area and the surrounding high-signal region (for the segmentation process see Figure 1).

The volume of the common vestibular nerve was measured—instead of the superior and inferior vestibular nerves separately—because these 2 branches are often not completely anatomically separated throughout the internal auditory canal. Anatomical studies have shown that the vestibular nerve branches into superior and inferior divisions only near the lateral end of the canal, typically at or just beyond the falciform crest. For most parts of their intrameatal course, these divisions are fused and cannot be distinctly visualized or separated, either in cadaveric dissection or by imaging. Because of this anatomical continuity, volumetric measurements are most reliable when assessing the common vestibular trunk, as attempting to distinguish and separately measure the individual branches would not be consistently feasible or reproducible (23, 24). As soon as the common vestibular trunk separated into its superior and inferior branches at the level of the fundus/falciform crest, the individual branch volumes were measured separately and then added to the proximal common trunk volume to obtain the total vestibular nerve volume.

#### 2.3.2 EH grading

**Figure 2:**
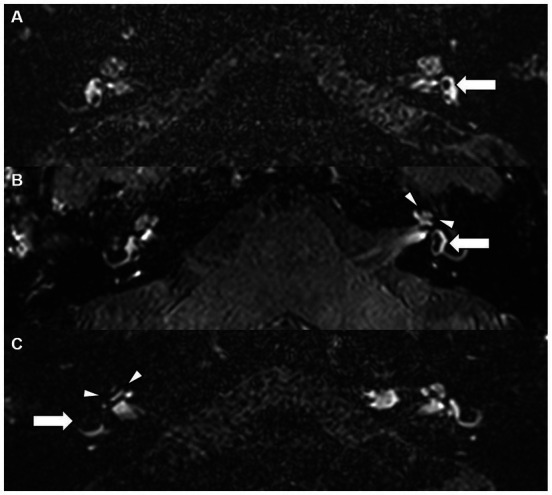
Examples of EH grading. Delayed post-gadolinium axial 3D T2 inversion recovery **(A,C)** and delayed post-gadolinium axial 3D FLAIR images. **(A)** Slight asymmetrical perilymphatic enhancement, more pronounced on the left side. There is enlargement of the saccule on the left (white arrow) without fusion with the utricle, consistent with a vestibular endolymphatic hydrops grade 1. Normal inner ear fluids in the left cochlea and on the right side. **(B)** Asymmetrical perilymphatic enhancement, more pronounced on the left. Nodular hypo-intensities in the periphery of the apical and midturn of the cochlea on the left (arrow heads). No bandlike hypo-intensities in the basal turn of the cochlea making this a cochlear hydrops grade 1. The left saccule is enlarged and is just confluent with the utricle with a remaining peripheral rim enhancement, making this a vestibular hydrops grade 2. **(C)** There is fusion of the utricle and saccule on the right and the perilymphatic enhancement is no longer visible (arrow), compatible with a vestibular hydrops grade 3. The scala vestibuli is fully obliterated due to the distended cochlear duct (arrow heads), cochlear hydrops grade 2.

EH grading for cochlear and vestibular EH was independently performed by 2 readers (AP and FS, a general radiologist with 10 years’ experience in general radiology) based on established MRI criteria, classifying ears into discrete grades (Grade 0/no EH, Grade 1, Grade 2, and Grade 3 vestibular EH; Grade 0/no EH, Grade 1 and Grade 2 cochlear EH), as described by Bernaerts et al. (25) (Figure 2). Disagreements between both readers were solved by a third reader (FW, European Diploma in Head and Neck Radiology holder with more than 20 years’ experience in head and neck and inner imaging). For grading vestibular EH, the saccule and utricle were assessed at the most inferior level of the vestibule to avoid artifactual fusion or apparent enlargement of these structures that can occur when evaluating more superior slices. All readers were blinded to clinical data. Control ears were those ears without any features of EH (Grade 0) on MRI according to any EH grading systems.

An example of EH grading is given in Figure 2.

### 2.4 Clinical data and symptom assessment

Data on clinical symptoms collected from patient records included presence or absence of hearing loss, vertigo, tinnitus, sudden hearing loss and aural fullness, recorded for each side.

Hearing loss was clinically documented by otolaryngologists at presentation (predominantly sensorineural). Specific pure-tone audiometry (PTA) results were not consistently available in medical records for full quantitative analysis, though clinical diagnosis followed standard otologic practice. Symptoms were referenced to the clinical status documented at the time of MRI referral.

### 2.5 Statistical analysis

Analyses were performed by JK. Data distributions were assessed for normality using Shapiro–Wilk tests. Due to non-normal distributions in most groups, non-parametric tests were predominantly used. The Kruskal-Wallis H-test assessed differences in nerve volumes across EH grades. Post-hoc pairwise comparisons employed Mann–Whitney U tests with Bonferroni correction.

To compare affected and unaffected ears in patients with unilateral hearing loss, paired t-tests were used when differences were normally distributed, supported by Wilcoxon signed-rank tests as needed.

Associations between EH severity (e.g., grades 2/3 vs. 1) and symptom prevalence were examined using chi-square tests, calculating odds ratios and effect sizes (Cramer’s V). Statistical significance was set at *α* = 0.05, with adjustments for multiple comparisons as applicable.

All analyses were performed using Python Version: 3.11.6; statsmodels 0.14.0; pandas 2.2.3; scikit-learn 1.6.1.

## 3 Results

### 3.1 Patient population

A total of 108 participants (mean age 53.6 years ± 15.8 years 95% CI, range: 20–85 years, 56.5% female and 43.5% male) were included in the study. Both ears were evaluated separately, yielding a total of 216 ears for analysis.

Most of the patients exhibited mild to moderate grades of EH. Specifically, grade 1 left cochlear EH was present in 42.6% of the patients, while grade 2 left and right vestibular EH were observed in 60.2 and 71.3% of patients, respectively.

Sensorineural hearing loss was the most prevalent clinical condition, affecting 70.4% of the cohort. Vertigo was also common, occurring in 69.4% of patients.

Mean nerve volumes were similar between the left and right sides for both cochlear and vestibular nerves, with average cochlear nerve volumes around 9–10 mm^3^ and common trunk vestibular nerve volumes approximately 19 mm^3^.

In summary, the dataset was characterized by a predominance of moderate-grade EH and a high prevalence of sensorineural hearing loss and vertigo (Table 2).

**Table 2:** Descriptive statistics.

Variable	*N*	Statistics
Demographics		
Sex	108	Female: 61 (56.5%), Male: 47 (43.5%)
Age at MRI (years)	108	53.6 years ± 15.8 (0.9–84.9)
Cochlear endolymphatic hydrops		
Left cochlear endolymphatic hydrops grade 1	108	20 (18.5%)
Left cochlear endolymphatic hydrops grade 2	108	46 (42.6%)
Right cochlear endolymphatic hydrops grade 1	108	21 (19.4%)
Right cochlear endolymphatic hydrops grade 2	108	37 (34.3%)
Nerve volumes (mm^3^)		
Left cochlear nerve volume	108	9.52 ± 4.92 (1.12–23.20)
Left common trunk vestibular nerve volume	108	18.95 ± 9.70 (4.32–49.50)
Right cochlear nerve volume	108	9.10 ± 5.90 (1.33–30.00)
Right common trunk vestibular nerve volume	108	19.78 ± 10.18 (2.50–48.70)
Vestibular endolymphatic hydrops		
Left vestibular endolymphatic hydrops grade 1	108	23 (21.3%)
Left vestibular endolymphatic hydrops grade 2	108	65 (60.2%)
Left vestibular endolymphatic hydrops grade 3	108	1 (0.9%)
Right vestibular endolymphatic hydrops grade 1	108	17 (15.7%)
Right vestibular endolymphatic hydrops grade 2	108	77 (71.3%)
Right vestibular endolymphatic hydrops grade 3	108	0 (0.0%)
Clinical conditions		
Sudden hearing loss	108	23 (21.3%)
Right-sided hearing loss	108	27 (25.0%)
Left-sided hearing loss	108	24 (22.2%)
Bilateral hearing loss	108	27 (25.0%)
Sensorineural hearing loss	108	76 (70.4%)
Conductive hearing loss	108	14 (13.0%)
Vertigo	108	75 (69.4%)
Tinnitus	108	48 (44.4%)
Aural fullness	108	19 (17.6%)

Demographic data, endolymphatic hydrops grading, cranial nerve volumes, and clinical features are shown for all patients included in the study (*N* = 108). Grading of endolymphatic hydrops is provided separately for left and right cochlear and vestibular structures. Clinical conditions include main auditory symptoms and vestibular complaints at presentation.

### 3.2 MRI assessment, volumetry, and EH grading

#### 3.2.1 Cochlear nerve volume by cochlear EH grade

Analysis of cochlear nerve volume across cochlear EH grades using the Kruskal-Wallis H-test did not demonstrate a statistically significant difference (*H* = 5.74, *p* = 0.057). Sample sizes included 91 ears without EH (Grade 0) (mean volume 8.38 ± 5.01 mm^3^), 41 ears with Grade 1 EH (10.68 ± 7.14 mm^3^), and 83 ears with Grade 2 EH (9.71 ± 4.73 mm^3^).

**Figure 3:**
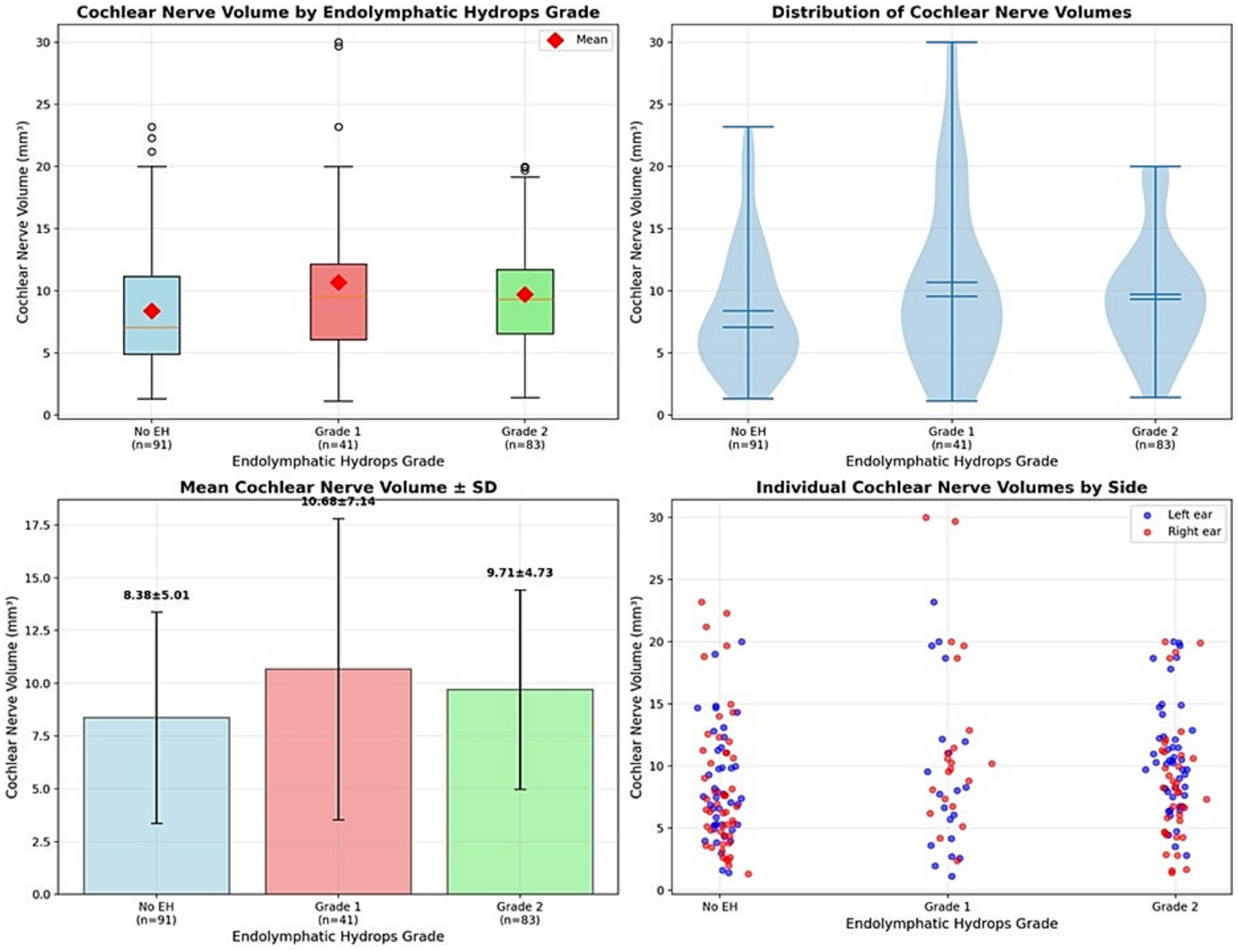
Cochlear nerve volume across cochlear endolymphatic hydrops grades. Boxplots illustrate cochlear nerve volumes (mm^3^) measured by MRI in ears classified by cochlear endolymphatic hydrops (EH) severity: No EH, Grade 1, and Grade 2. The sample size was *n* = 91 (no EH), *n* = 41 (Grade 1), and *n* = 83 (Grade 2). Median volumes and interquartile ranges are shown. Although a tendency toward larger nerve volumes is observed in patients with Grade 1 EH compared to no EH, no statistically significant difference was detected after Bonferroni correction (Kruskal-Wallis *H* = 5.74, *p* = 0.057). One ear with Grade 3 EH was excluded due to insufficient data.

Post-hoc pairwise comparisons using Mann–Whitney U tests indicated a nonsignificant tendency toward increased cochlear nerve volume in participants with Grade 1 compared to no EH (*p* = 0.023). However, this did not remain significant after Bonferroni correction (adjusted *α* = 0.0167). No other pairwise comparisons reached significance. These results suggest that there is no robust association between cochlear EH severity and cochlear nerve volume (Figure 3).

#### 3.2.2 Vestibular nerve volume by vestibular EH grade

**Figure 4:**
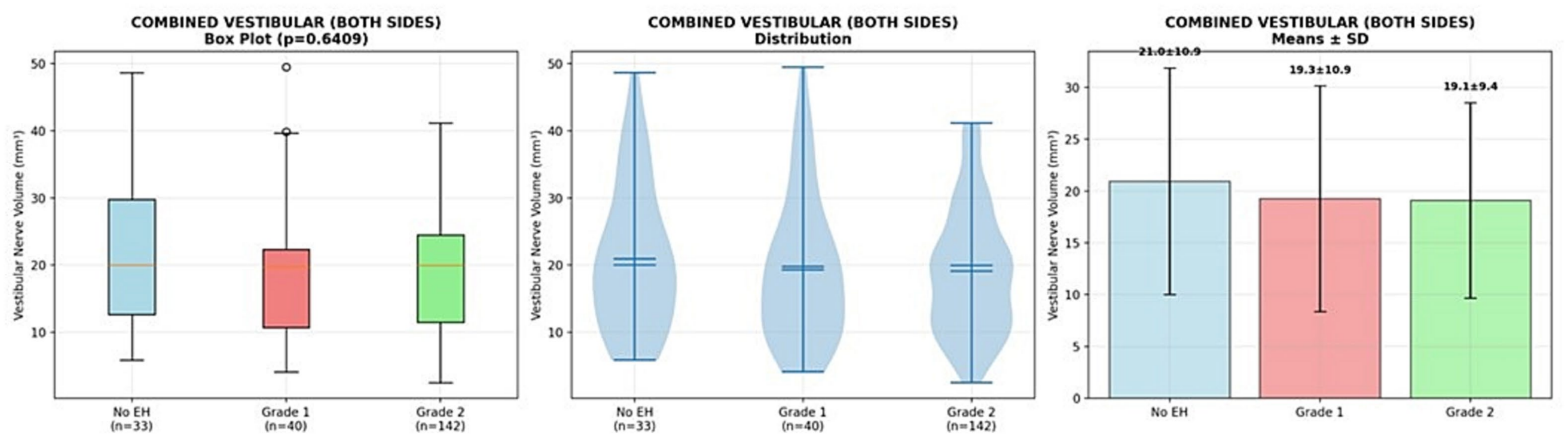
Vestibular nerve volume across vestibular endolymphatic hydrops grades. Boxplots represent vestibular nerve volumes (mm^3^) obtained from MRI, categorized by vestibular endolymphatic hydrops (EH) grades: no EH (*n* = 33), grade 1 (*n* = 40), and grade 2 (*n* = 142). Data distributions substantially overlap, and statistical analysis showed no significant differences between groups (Kruskal-Wallis *p* = 0.64).

Similarly, vestibular nerve volumes did not differ significantly by vestibular EH grade (Kruskal-Wallis *p* = 0.641). Sample sizes included 33 ears with no vestibular EH (20.96 ± 10.93 mm^3^), 40 with Grade 1 (19.27 ± 10.88 mm^3^), and 142 with Grade 2 (19.10 ± 9.43 mm^3^). Pairwise comparisons indicated no significant differences after correction. Thus, vestibular nerve volume appears stable regardless of vestibular EH severity (Figure 4).

#### 3.2.3 Comparison of cochlear nerve volumes in affected versus unaffected ears

In 51 patients with unilateral hearing loss, paired comparison of cochlear nerve volume between affected and unaffected ears revealed no significant difference (paired *t*-test *t* = 0.581, *p* = 0.564; Wilcoxon signed-rank test *p* = 0.390). The negligible mean volume difference of 0.57 mm^3^ and small effect size (Cohen’s *d* = 0.081) suggest that no significant differences in cochlear nerve caliber are associated with unilateral auditory impairment.

#### 3.2.4 Associations between EH severity and clinical symptoms

Chi-square tests assessing the relationship between EH severity and symptom prevalence (hearing loss, vertigo, tinnitus, and aural fullness) failed to find statistically significant associations for either cochlear or vestibular EH grades. The most related clinical findings, albeit nonsignificant, included left-sided severe cochlear EH with vertigo (*p* = 0.060) and hearing loss (*p* = 0.067), as well as left-sided severe vestibular EH with aural fullness (*p* = 0.107). Effect sizes were small to very small, and odds ratios had wide CIs encompassing 1.0, indicating lack of reliable predictive associations.

## 4 Discussion

### 4.1 Study focus and rationale

This study evaluated the impact of EH severity on cochlear and vestibular nerve volume using high-resolution MRI and explored potential correlations with clinical symptoms in a sizable cohort. It addresses whether volumetric MRI of the vestibulocochlear nerve can serve as a robust imaging biomarker of EH.

### 4.2 Comparison with previous findings on cochlear nerve alterations

The earlier study by Khalifa et al. reported a significant reduction in cochlear nerve caliber associated with EH consistent with the neural loss hypothesis supported by animal studies. Megerian et al. showed that induced EH in guinea pigs led to reduced cochlear nerve diameter, reflecting neuronal injury (26). Our findings did not replicate this reduction in nerve volume, suggesting that measurable nerve atrophy may not occur—or may occur only in advanced disease stages. In our cohort, both cochlear and vestibular nerve volumes remained largely stable across EH severities, with only nonsignificant tendencies in cochlear volume in milder grades. This divergence emphasizes that volumetric stability may itself hold pathological relevance, pointing toward early compensatory or non-neuronal mechanisms underlying EH. Methodological distinctions and implications for interpretation.

We employed volumetric segmentation capturing the entire nerve in three dimensions, whereas Khalifa et al. (5) used two-dimensional cross-sectional area measures. Cross-sectional area measurements are often limited by variability in the imaging plane and partial volume effects, which may lead to under- or overestimation of nerve size, especially in small nerves with complex morphology. Volumetry’s sensitivity to diffuse or irregular changes along the nerve may yield fundamentally different interpretative scales, focusing more on global preservation than focal thinning. Cross-sectional techniques may also be susceptible to slice orientation and partial volume effects, potentially explaining apparent discrepancies.

By using a semi-automated segmentation workflow and adjusting for multiple comparisons, our analysis prioritizes robustness and reproducibility. The absence of significant results therefore strengthens confidence that large-scale volumetric nerve loss is unlikely to represent a consistent hallmark of EH.

### 4.3 Integrating vestibular findings

Wang et al. described ultrastructural degeneration in the vestibular nerve linked to progressive vertigo (27). While this may support a vestibular neurodegeneration hypothesis, our volumetric findings did not identify correlating structural loss, suggesting either an earlier disease stage or that such degeneration occurs below the resolution of current MRI. The preserved nerve volumes observed here may indicate that measurable morphological changes in both cochlear and vestibular nerves develop later than functional or biochemical alterations.

### 4.4 Relation to previous facial and vestibulocochlear nerve findings

Our results also contrast with those of Henneberger et al., who reported swelling of the facial and vestibulocochlear nerves within the study group when compared to the control group of participants with normal hearing (28) Again, important methodological distinctions may explain this discrepancy. Henneberger et al. applied manual tracing on single axial slices to estimate nerve cross-sectional area, whereas our study used volumetric segmentation of nerves along their entire length within the internal auditory canal, providing a comprehensive three-dimensional measurement. We interpret these apparent enlargements as possible transient or inflammatory responses rather than markers of chronic neurodegeneration, given that volumetric analysis across the entire nerve is less influenced by focal edema. The analogous facial nerve findings may instead align with molecular hypotheses emphasizing mediator-driven rather than degenerative mechanisms (2, 4, 8, 10, 29–31).

### 4.5 Interpretation and implications of negative findings

Although nerve volumes did not differ across EH grades, this outcome refines the field’s understanding of disease mechanisms. It suggests that endolymphatic pressure alterations and symptom generation likely stem from processes other than overt volumetric nerve changes; such as synaptic disruption, ion channel dysfunction, or altered protein composition within the inner ear fluids (10, 29–31). These findings highlight the need for multimodal approaches combining functional, molecular, and volumetric imaging to fully capture disease expression.

### 4.6 Clinical associations and future directions

The lack of correlation between EH severity and clinical features—hearing loss, vertigo, tinnitus, and aural fullness—underscores the multifactorial nature of symptom production. This clinical dissociation mirrors our anatomical observations, where cochlear and vestibular nerve volumes remained stable regardless of EH grade. Maihoub et al. evaluated 43 definite MD patients using audiometry and caloric testing, finding no correlation between hearing loss stage and canal paresis. They concluded that audiometric changes do not directly correspond with the vestibular ones; therefore, no specific correlation exists between them (32). This supports the hypothesis that EH pathophysiology affects cochlear and vestibular divisions through distinct mechanisms, or that both functional and structural changes may require longer disease duration or advanced MRI techniques to become detectable. This aligns with the concept that early EH may be primarily driven by reversible or dynamic mechanisms, preceding measurable structural degeneration. Future studies should incorporate longitudinal imaging, biochemical profiling, and functional testing to identify early biomarkers and disease subtypes.

### 4.7 Limitations

Some limitations of this study warrant consideration. First, the retrospective design precluded formal Bárány classification of Ménière’s disease due to incomplete historical symptom documentation. Secondly, symptoms were referenced to the clinical status documented at the time of MRI referral rather than prospectively assessed at the exact time of imaging. Consequently, symptom documentation reflects the clinical status proximal to imaging but may not fully capture the episodic fluctuations typical of MD. Although our cohort represents one of the largest MRI-based studies of EH to date (*n* = 108), all patients were recruited from a single tertiary center in Switzerland. This regional homogeneity may limit generalizability to more diverse populations with varying genetic, environmental, or disease-duration profiles.

Even with high-resolution 3D volumetry, current MRI techniques cannot resolve microstructural or synaptic-level changes within cranial nerves, which may precede detectable volumetric alterations. Subtle axonal degeneration, synaptic loss, or inflammatory processes thus remain beyond the scope of this imaging approach. Future studies should extend these observations using longitudinal designs to monitor nerve volume changes correlated with disease progression and symptom fluctuations. Incorporating advanced imaging modalities that assess nerve microstructure (e.g., diffusion tensor imaging) and functional metrics may yield deeper insights into the pathophysiology of EH and MD.

## 5 Conclusion

In summary, our study indicates that EH severity is not associated with significant changes in cochlear or vestibular nerve volumes detectable by MRI, nor is it strongly predictive of clinical symptoms. This suggests that cochlear and vestibular nerve volumetry should not be considered a reliable imaging biomarker of EH at present. Future research should prioritize alternative MRI biomarkers—such as perilymphatic signal alterations, endolymphatic sac metrics, and microstructural diffusion techniques—to better elucidate the pathophysiology of hydropic ear disease.

## Data Availability

The raw data supporting the conclusions of this article will be made available by the authors, without undue reservation.
